# Are there gap junctions without connexins or pannexins?

**DOI:** 10.1186/s12862-019-1369-4

**Published:** 2019-02-26

**Authors:** Georgy A. Slivko-Koltchik, Victor P. Kuznetsov, Yuri V. Panchin

**Affiliations:** 1 Kharkevich Institute for Information Transmission Problems, Russian Academy of Sciences, Moscow, Russian Federation 127994; 2 A.N. Belozersky Institute of Physico-Chemical Biology Moscow State University, Moscow, Russian Federation 119991

**Keywords:** Connexin, Pannexin, Innexin, Gap junctions, Intercellular channels

## Abstract

**Background:**

Gap junctions (GJ) are one of the most common forms of intercellular communication. GJs are assembled from proteins that form channels connecting the cytoplasm of adjacent cells. They are considered to be the main or the only type of intercellular channels and the universal feature of all multicellular animals. Two unrelated protein families are currently considered to be involved in this function, namely, connexins and pannexins (pannexins/innexins). Pannexins were hypothesized to be the universal GJ proteins of multicellular animals, distinct from connexins that are characteristic of chordates only. Here we have revised this supposition by applying growing high throughput sequencing data from diverse metazoan species.

**Results:**

Pannexins were found in Chordates, Ctenophores, Cnidarians, and in the most major groups of bilateral protostomes. Yet some metazoans appear to have neither connexins nor pannexins in their genomes. We detected no connexins or pannexins/innexins homologues in representatives of all five classes of echinoderms and their closest relatives hemichordates with available genomic sequences. Despite this, our intracellular recordings demonstrate direct electrical coupling between blastomeres at the 2-cell embryo of the echinoderm (starfish *Asterias rubens*). In these experiments, carboxyfluorescein fluorescent dye did not diffuse between electrically coupled cells. This excludes the possibility that the observed electrical coupling is mediated by incomplete cytoplasm separation during cleavage.

**Conclusion:**

Functional GJs are present in representatives of the clade that lack currently recognized GJ protein families. New undiscovered protein families utilized for intercellular channels are predicted. It is possible that the new type(s) of intercellular channels are present in parallel to pannexin and connexin gap junctions in animal groups, other than Echinodermata.

## Introduction

**Fig. 1:**
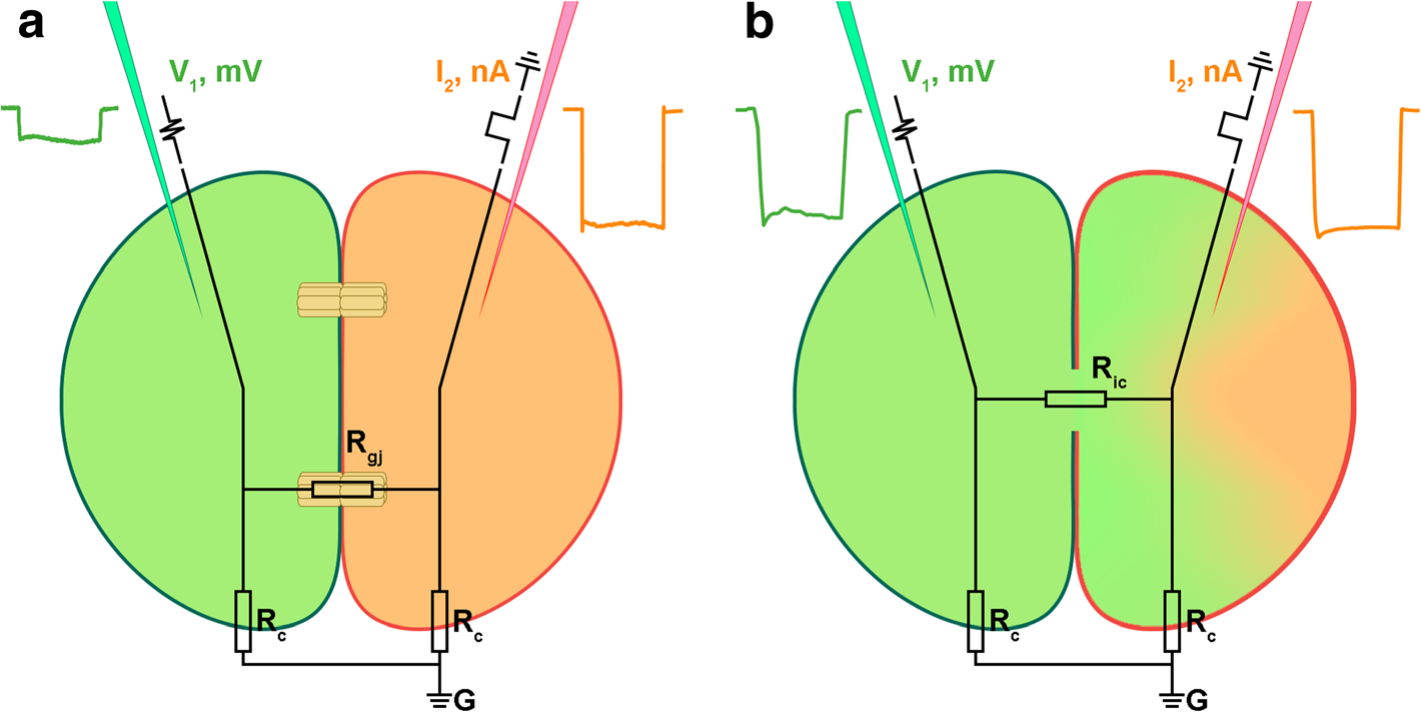
Gap junctions versus syncytium or incomplete cleavage. **a** Cells connected by GJs are electrically coupled via intercellular channels. Electrical current pulse applied through intracellular microelectrode I_2_ results in a small ion molecule flow through GJs and leads to membrane potential changes in the adjacent cell recorded by electrode V_1_. If tracer dye ejected from V_1_ electrode (green) is bigger than the GJ channel pore size, it is restricted to the cytoplasm of one cell. **b** Incompletely divided or fused cells show a voltage drop on shared plasma membranes in response to the electrical current pulse applied via I_2_ (R_ic_ < < R_gj_) and at the same time big molecules freely diffuse through the shared cytoplasm

Intercellular communication is the basis for coordinated function of multicellular organisms. Numerous types of molecules were shown to transfer biological signals from one cell to another. Typically, such molecules are released by a special mechanism from one cell and diffuse into the extracellular space to act on the neighboring or distant cells of the same multicellular organism. Another route of communication requires direct cellular contacts that may use signal molecules and their receptors, bound to membranes of the adjacent cells. The most intimate intercellular contacts occur by the direct linkage of the cytoplasm of two cells. In some cases, complete cell fusion may be similar to the multinucleated syncytia [1]. Otherwise, distinct specialized channels called gap junctions (GJs) may link adjoining cells [2–4]. Intercellular channels in multicellular animals were first discovered as electrical synapses between neurons [5]. Establishment of the electrical coupling between adjacent cells with the electrophysiological techniques shaped the basis of GJ studies [1, 5, 6]. Later it was found that these channels are permeable not only to small ions but in some cases to bigger molecules including fluorescent tracer dyes that allow the detection of GJs and the estimation of channel size and properties [1, 6–8]. Fluorescent tracer dyes are also used to distinguish syncytium from proteinaceous GJ channels [6, 9, 10]. In the case of cells fused in syncytium or incompletely separated cells upon the cell division event even big hydrophilic molecules readily spread to the cytoplasm of all connected cells but are restricted to individual cells connected by GJ (Fig. 1).

Connexins were identified as the molecular components of vertebrate GJ about 30 years ago [11]. For a long time it was assumed that connexins were the only family of GJ proteins. As long as numerous attempts to clone connexins from invertebrates failed, it was finally suggested that invertebrate GJ are assembled from proteins unrelated to connexins [12]. This protein family was found in studies that analyzed *Drosophila* and *C. elegans* mutants, and was originally designated OPUS for four founder protein family members: **o**gre, **p**assover, **u**nc-7 and **s**hakingB [12–15]. Later they were renamed innexins, suggesting that they were specific invertebrate GJ proteins [16]. Fifteen years ago, we found the presence of innexin homologues in humans and other vertebrates, and proposed to reclassify innexins and their vertebrate homologues into a bigger family, named pannexins [17–19]. In this paper, we will use the terms innexins, pannexins or innexins/pannexins as synonymous.

Analysis of currently available massive high throughput sequencing genomic and transcriptomic data allows us to understand not only what genes are present in a given organism but also to pinpoint genes that are absent in the genome. The most intriguing outcome of such survey for GJ proteins was the absence of both GJ protein families in several species despite the general view, where GJ are universal in all animals. Previously we reported that sea urchins (*Strongylocentrotus purpuratus*) are devoid of both connexins and pannexins [9, 10, 20, 21]. The list of such species has now grown and includes Sponges, Trichoplax, Echinoderms, Tardigrades, Priapulids and several other animals (see results section).

There are almost no publications on functional GJ studies on species lacking both connexins and pannexins. The only exceptions are three papers related to low electrical resistance cell junctions on two echinoderm species [22–24]. These studies were performed prior to pannexins discovery and sea urchin and other echinoderm genome sequencing. Additionally authors did not use tracer dyes [22, 23] or used dyes for which GJ were permeable making discrimination between cytoplasmic bridges and GJ channels complicated [24]. In this paper, we first screened available diverse high throughput sequencing genomic and transcriptomic data from different metazoans for connexin and pannexin orthologs and found several groups of animals that lack both protein families in their genomes including twelve species of Echinoderms. Next, using electrophysiological techniques combined with intracellular dye injections, we demonstrated functional GJs in the Echinoderm *Asterias rubens* 2-cells embryo.

## Material and methods

**Fig. 2:**
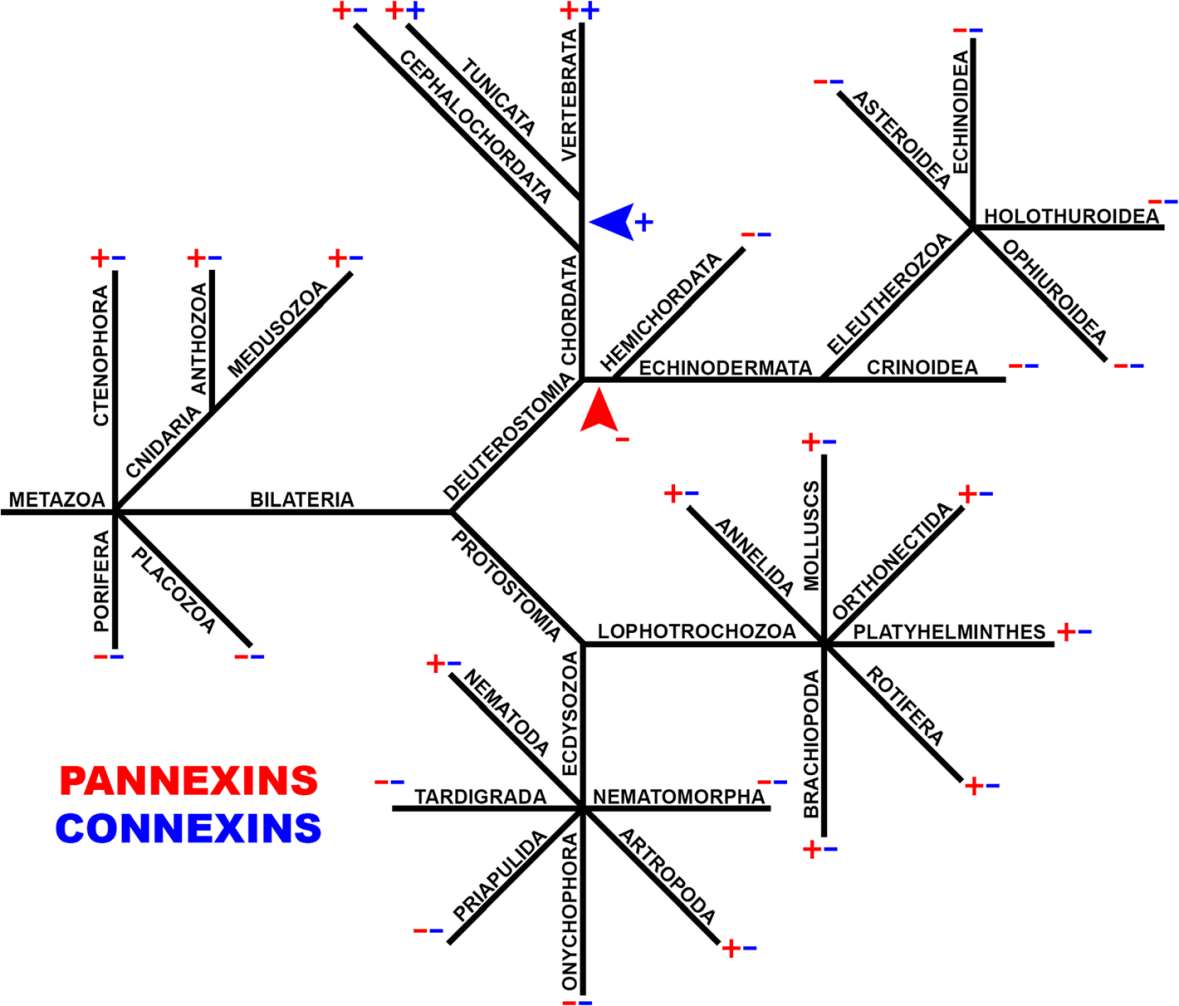
GJ proteins evolution. Connexin (blue) and pannexin (red) presence (+) or absence (−) in the multicellular animal main taxonomic groups is indicated in simplified phylogenetic tree. The blue arrowhead points out a hypothetical connexin acquisition event in Chordate evolution. The red arrowhead shows a hypothetical event of pannexin loss at the base of Echinodermata/Hemichordata’s sister group branch

Using HMMER (http://hmmer.org/) we searched for Innexin (PF00876) and Connexin (PF00029) PFAM domains from eukaryote databases at NCBI (https://www.ncbi.nlm.nih.gov/) and PFAM (https://pfam.xfam.org/). GJ proteins from different species were taken to perform systematical search via BLASTP and TBLASTN in individual organisms or big phylogenetic groups. For connexins 10 assorted query proteins were taken from tunicates and chordates and for innexins/pannexins 20 assorted query proteins were taken from ctenophores, cnidarians, arthropods, nematodes, mollusks and chordates (Fig. 2).

Common starfish *Asterias rubens* were collected at The White Sea Biological Station “Kartesh” (WSBS) of the Zoological Institute of Russian Academy of Sciences (Saint-Petersburg). Gametes were obtained by dissection. Oocytes were fertilized in vitro. Experimental embryos were recorded in seawater at 12 °C in a chamber with 300 μm plastic wells on its bottom. Axoclamp 2B amplifiers (Axon Instruments) and 2 M KCl-loaded glass microelectrodes with 20–40 MΩ resistance were used for recordings. Current/voltage signals were acquired and analyzed using DigiData 1200 series ADC and pCLAMP software (Molecular Devices, Sunnyvale, CA, USA). SigmaPlot 9.0 software (Systat Software, Inc., San Jose, CA, USA) and Microsoft Excel 2013 were used for statistical analysis. For dye injections one electrode was filled with 3% carboxyfluorescein (Sigma 21,877) in 0.2 M KCl. Negative current up to 12 nA (I_2_ electrode) was injected into single blastomeres of the 2-cell stage embryos (Fig. 1). In all experiments, one blastomere was ionophoretically injected by carboxyfluorescein dye through the V_1_ electrode. Injected embryos were visualized and photographed with an Olympus BX51 luminescent microscope and an Olympus XC10 digital camera. The filter set was optimized for fluorescein.

**Fig. 3:**
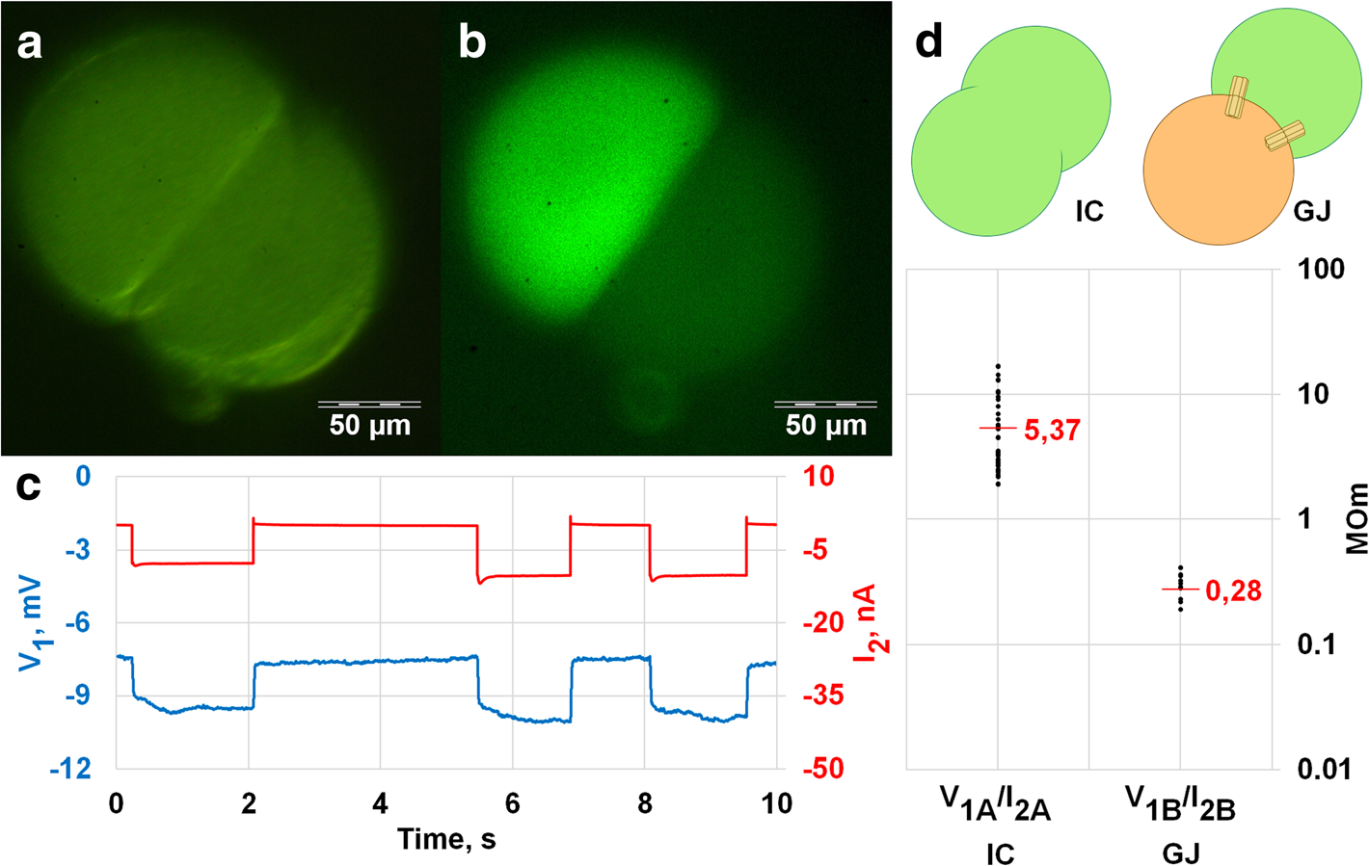
Electrical coupling in *A. rubens* early embryos. **a**-**c** Microelectrode V_1_ placed in one blastomere registers the membrane potential changes, whereas I_2_ electrode inside the adjacent blastomere is used to inject current pulses. **a**, **b** Fluorescent dye applied via V_1_ electrode stain only injected cell . Plain light background **a** and fluorescence image **b** of 2-cell embryo. **c** Negative current impulses (red) are applied to test electrical coupling. Electrical potential changes (blue) are recorded via V_1_ electrode. (a, b and c are from the same experiment). **d**. V_1_/I_2_ values in two different situations from 51 current impulse experiments. The difference between IC and GJ is significant (*p*-value< 0.05). On the left (A) V_1_/I_2_ value was measured in incompletely cleaved zygote and corresponds to cell input resistance R, on the right (B) V_1_/I_2_ was measured in separated blastomeres

According to Ohm’s Law the approximate value of GJ resistance R_gj_ was calculated by the equation based on the simple electrical circuit depicted in Fig. 1 with a reasonable assumption that the total membrane resistance does not change upon cleavage, blastomeres are identical and R_ic_ ≈ 0.

$$ {\displaystyle \begin{array}{l}{R}_{gj}=\frac{I_{c2B}{R}_{c2B}}{I_{c1B}}-{R}_{c1B}=\frac{\left({I}_{2B}-\frac{V_{1B}}{R_{c1B}}\right){R}_{c2B}}{\frac{V_{1B}}{R_{c1B}}}-{R}_{c1B}=\frac{I_{2B}{R^2}_c-{V}_{1B}{R}_c}{V1B}-{R}_c=\frac{I_{2B}{R^2}_c}{V_{1B}}-\\ {}2{R}_c={\left(\frac{V_{1B}}{I_{2B}}\right)}^{-1}{(2R)}^2-2(2R)=4\left({\left(\frac{V_{1B}}{I_{2B}}\right)}^{-2}{\left(\frac{V_{1A}}{I_{2A}}\right)}^2-\left(\frac{V_{1A}}{I_{2A}}\right)\right),\end{array}} $$

where the A index is used, for incomplete cleaved zygote experiments while the B index for experiments with 2 blastomeres connected by gap junctions (Fig. 3d).

## Results and discussion

### Loss and acquisition of GJ proteins in Metazoa

Our updated search for connexins and innexins/pannexins in protein and nucleotide databases via BLAST and HMMER (http://hmmer.org/) confirmed an earlier observation [20, 21] that indicates no homologues of these proteins in cellular organisms outside Metazoa clade. Figure 2 shows a simplified phylogenetic tree of metazoans, which points out connexin (blue) and pannexin (red) presence or absence in the main taxonomic groups. Research confirms that connexins are universal for craniata (vertebrates) and tunicates, whereas no connexins could be found in the basal cephalochordate (lancelets) branch of chordate evolution. This suggests a relatively late acquisition of the new GJ proteins (connexins) in the background of earlier pannexin presence. In basal metazoans, pannexins are present in Ctenophores and Cnidarians but were not found in Placozoa and Porifera. Species from two related groups of extremely simplified parasitic Cnidarians – Myxozoa and Polypodiozoa (*Polypodium hydriforme*) also appear to lack pannexins.

Unexpectedly we were not able to detect pannexin homologues in several protostome Ecdysozoa phyla: Tardigrada (*Hypsibius dujardini*; *Ramazzottius varieornatus*) Nematomorpha (*Paragordius varius*), Onychophora (*Euperipatoides rowelli*) and Priapulida (*Priapulus caudatus).* Previously it was stated that both connexin and pannexin genes are absent in the sea urchin genome [9, 10, 20, 21, 25]. Genome sequencing analysis confirms this conclusion [26].

Recently, several additional species from different Echinodermata orders (Crinozoa, Asterozoa, Echinozoa, Blastozoa, Crinoidea) were sequenced and added to NCBI database. Eleven available Echinodermata genomes (*Ophiothrix spiculata, Apostichopus japonicus, Acanthaster planci, Eucidaris tribuloides, Lytechinus variegatus, Patiria miniata, Hemicentrotus pulcherrimus, Strongylocentrotus purpuratus, Ophionereis fasciata, Apostichopus parvimensis* and *Patiriella regularis)* and one SRA experiment (Crinoidea, *Antedon mediterranea*) were investigated and we have found no connexin or pannexin homologues in this data (Table 1). GJ genes were also not found in Hemichordates (*Ptychodera flava* and *Saccoglossus kowalevskii*) another deuterostome phylum, generally considered the sister group of the echinoderms.

**Table 1:** Presence (+) or absence (−) of connexins and pannexins in the main taxonomic groups of multicellular animals. Lack of both types of GJ was found in some taxonomic groups: Echinodermata (12 spicies), Hemichordata(2), Tardigrada(2), Nematomorpha(1), Onychophora(1)

	Taxon	Species	Pannexins	Connexins
Deuterostomia	Vertebrata	*Homo sapiens*	+	+
	Tunicata	*Ciona intestinalis*	+	+
	Cephalochordata	*Branchiostoma lanceolatum*	+	–
	Echinodermata	Ophionereis fasciata	–	–
		Patiriella regularis	–	–
		*Strongylocentrotus purpuratus*	–	–
		Apostichopus parvimensis	–	–
		Hemicentrotus pulcherrimus	–	–
		*Ophiothrix spiculata*	–	–
		*Apostichopus japonicus*	–	–
		*Acanthaster planci*	–	–
		*Eucidaris tribuloides*	–	–
		*Lytechinus variegatus*	–	–
		*Patiria miniata*	–	–
		Antedon mediterranea	–	–
	Hemichordata	Ptychodera flava	–	–
		*Saccoglossus kowalevskii*	–	–
Protostomia	Plathelminthes	Hymenolepis diminuta	+	–
	Orthonectida	*Intoshia linei*	+	–
	Annelida	Capitella teleta	+	–
	Brachiopoda	*Lingula anatina*	+	–
	Molluscs	*Aplysia californica*	+	–
	Acanthocephala	*Echinorhynchus gadi*	+	–
	Bryozoa	*Membranipora membranacea*	+	–
	Cycliophora	*Symbion pandora*	+	–
	Entoprocta	*Loxosoma pectinaricola*	+	–
	Nematoda	*Caenorhabditis elegans*	+	–
	Arthropoda	*Drosophila melanogaster*	+	–
	Tardigrada	*Hypsibius dujardini*	–	–
		*Ramazzottius varieornatus*	–	–
	Nematomorpha	*Paragordius varius*	–	–
	Onychophora	*Euperipatoides rowelli*	–	–
	Ctenophora	*Pleurobrachia bachei*	+	–
	Cnidaria	Hydra vulgaris	+	–

The views on early metazoan evolution are controversial [27] and the observed GJ gene existence pattern could either indicate the basal absence of pannexins in Porifera, and their eventual loss in Placozoa or a simultaneous pannexin loss in both Placozoa and Porifera, if Ctenophores are more basal metazoans. Anyhow, the presence of pannexins in the base of the metazoan tree allows us to interpret pannexin absence in some bilaterians as a gene loss.

The set of GJ proteins varies in different species with several loss and acquisition events. In extant species, GJ protein genes are present in multiple copies. For humans and model animals, it was shown that GJs are essential for organism fitness and survival, however evolutionary loss of such important genes is not clearly explained [28–31]. Phylogenomic trees show that pannexins from different big metazoan phyla are not intermixed in tree nodes and the generated tree satisfies commonly accepted metazoan taxonomy [32–34]. This observation indicates that only one precursor pannexin gene was acquired vertically from the common ancestor and then was then diversified independently in each animal phylum. The evolutionary loss of a single gene is more possible than the consequent loss of many genes from an important protein family. The reduction of the pannexin family can be associated with the competition versus newly acquired connexins. Similarly, the loss of pannexins might be simplified when another protein substitutes for them in GJ function.

### Intercellular channels in *Asterias rubens*

The existence of animals lacking both connexins and pannexins in their genomes challenges common views on intercellular channels. Unfortunately, there are almost no functional GJ studies on such species. Preferably, the presence of GJ type of intercellular channels has to be supported by direct electrophysiological cell coupling measurements. Together with intracellular tracer dye injections, it allows us to make a distinction between real GJ channels and incomplete cytoplasm separation or cell fusion (Fig. 1). These kinds of experiments require the use of relatively large cells which allows us to impale at least two microelectrodes or patch pipettes in adjacent cells. For instance, it is possible to take an advantage of big cells of the early embryo. Blastomeres are much bigger than developed cells allowing intercellular electrical coupling to be measured by conventional electrophysiological methods [35, 36]. In several invertebrate GJ studies, it was shown that carboxyfluorescein fluorescent dye did not diffuse between electrically coupled cells [9, 10, 37]. This dye is membrane-impermeant and can be easily ejected from glass microelectrodes electroforetically. Therefore, it was selected for our electrophysiological experiments on the early two blastomere development stage of common starfish *Asterias rubens* embryo.

Two glass microelectrodes, one loaded with KCl and one filled with 3% carboxyfluorescein dissolved in KCl solution, were injected into two adjacent blastomeres right after presumed first zygote cleavage. To detect the electrical coupling short negative current pulses were applied through one electrode (Fig. 1b) while membrane potential changes were recorded by the other electrode. Voltage shift, measured by the second electrode indicates electrical coupling. Voltage drop measured by the microelectrode placed in the space between fertilization membrane and cell membrane in response to current pulse into the intracellular electrode placed in cytoplasm was below detectable values.

Fluorescent dye was injected from one electrode before electrical measurements to determine incomplete zygote cleavage. Electrical coupling between two electrodes was observed after cell membrane penetration in 14 *A.rubens* early embryos. In 10 experiments fluorescent dye ejected from one electrode evenly loaded the whole embryo of an apparently incompletely cleaved zygote. In 4 experiments fluorescent dye diffusion was confined to one blastomere (Fig. 3a, b). These separated cells were electrically coupled as shown in Fig. 3c. Figure 3d show V_1_/I_2_ values for two different situations. On the left (IC), V_1_/I_2_ is actually the membrane input resistance (R) of an incompletely cleaved zygote (inset on the top). On the right (GJ), we show the ratio of membrane voltage shift from blastomere 1 (V_1_) in response to a current pulse into a separate blastomere 2 to injected current pulse I_2_. Strict coupling coefficient or GJ resistance measurements between two cells require 3 or 4 electrodes with two electrodes (one for current and one for voltage) inserted in the same cell. Early starfish embryos are fragile and we were not able to use more than two electrodes without fatal cell damage. Yet with reasonable assumption listed in methods section we can estimate R_gj_ in the electrical circuit depicted in Fig. 1 as about 390 MΩ.

Thus, we conclude that starfish embryonic cells are connected by channels permeable for ions, but with a pore size small enough, to block soluble carboxyfluorescein molecules from passing. Our data is consistent with the earlier publications that reported low electrical resistance cell junction presence in two echinoderm species [22–24].

### Are there additional GJ proteins in Metazoa?

Our comparative genomic studies suggest that some metazoans appear to have neither connexins nor pannexins. This implies that, either some multicellular animals have no gap junctions, or an additional family(s) of GJ proteins may exist. Already published reports [22–24] and our new physiological data shows the presence of functional GJs in Echinoderms.

New type(s) of intercellular channels might be present in parallel to pannexin and connexin based gap junctions in several animal groups, other than Echinodermata. Some evidence in support of this hypothesis could be found in nematode studies. In a recent high-resolution expression study of all members of the pannexin (innexin) protein family in *Caenorhabditis elegans*, no detectable pannexin expression was found in some cells with well documented gap junctions [38–40].

## Conclusions

Twenty years ago, the existence of gap junction proteins other than connexins had not been merely unknown, but entirely unpredicted and unsuspected. Then the OPUS-innexin-pannexin family entered the stage. Although, previously we ourselves named pannexins, in opposite to Chordate-specific connexins, considering them as the universal metazoan GJ proteins, now we admit a possible future discovery of additional protein families utilized for GJ function.

## Abbreviation

GJ: Gap junctions
